# Impact of global warming at the range margins: phenotypic plasticity and behavioral thermoregulation will buffer an endemic amphibian

**DOI:** 10.1002/ece3.1315

**Published:** 2014-11-10

**Authors:** Manuel Ruiz-Aravena, Avia Gonzalez-Mendez, Sergio A Estay, Juan D Gaitán-Espitia, Ismael Barria-Oyarzo, José L Bartheld, Leonardo D Bacigalupe

**Affiliations:** 1 School of Biological Sciences, Faculty of Sciences, Engineering & Technology, University of TasmaniaHobart, Tasmania, Australia; 2 Facultad de Ciencias del Mar, Universidad Católica del NorteCoquimbo, Chile; 3 Instituto de Ciencias Ambientales y Evolutivas, Facultad de Ciencias, Universidad Austral de ChileCasilla 567, Valdivia, Chile

**Keywords:** Amphibian, behavioral thermoregulation, global warming, operative temperature, phenotypic plasticity, thermal performance curve, thermal tolerance

## Abstract

When dispersal is not an option to evade warming temperatures, compensation through behavior, plasticity, or evolutionary adaptation is essential to prevent extinction. In this work, we evaluated whether there is physiological plasticity in the thermal performance curve (TPC) of maximum jumping speed in individuals acclimated to current and projected temperatures and whether there is an opportunity for behavioral thermoregulation in the desert landscape where inhabits the northernmost population of the endemic frog *Pleurodema thaul*. Our results indicate that individuals acclimated to 20°C and 25°C increased the breath of their TPCs by shifting their upper limits with respect to when they were acclimated at 10°C. In addition, even when dispersal is not possible for this population, the landscape is heterogeneous enough to offer opportunities for behavioral thermoregulation. In particular, under current climatic conditions, behavioral thermoregulation is not compulsory as available operative temperatures are encompassed within the population TPC limits. However, for severe projected temperatures under climate change, behavioral thermoregulation will be required in the sunny patches. In overall, our results suggest that this population of *Pleurodema thaul* will be able to endure the worst projected scenario of climate warming as it has not only the physiological capacities but also the environmental opportunities to regulate its body temperature behaviorally.

## Introduction

The biodiversity of the earth is undergoing an extraordinary transformation as a result of the effects of human activities on every ecosystem (Vitousek 1992, 1994; Mooney and Cleland 2001). Although land change use still is the main driver of biodiversity loss and habitat fragmentation, without a doubt, global warming is projected to be the largest human-induced disturbance placed on natural ecosystems (Millenium Ecosystem Assessment 2005; Pereira et al. 2010; Beaumont et al. 2011).

The impact of current global warming on biodiversity has been widespread and has involved several types of responses (Parmesan 2006; Chown et al. 2010; Hoffmann and Sgro 2011). In overall, four compensatory mechanisms are possible for a population (or a species) in the face of warming to prevent extinction. Mobile species might migrate, given the structure of the landscape, to more favorable thermal environments tracking their current bioclimate envelope. If the thermal environment is heterogeneous, then mobile species might regulate their body temperature behaviorally (Kearney et al. 2009). If dispersal is not possible and/or if the thermal environment is rather homogeneous, then a population may adjust to a warming climate by physiological plasticity, or evolutionary adaptation (Huey et al. 2012).

Environmental temperature (T_a_) is the abiotic factor with major incidence in the physiology and ecology of most of biodiversity in the planet and this is particularly true for ectotherms (Angilletta 2009 and references therein). T_a_ plays a large role in determining their body temperature (T_b_) and the rate of their physiological processes (Hochachka and Somero, 2002; Young et al., 2011). This means that any performance trait (e.g., growth, reproduction, physiology) in an ectothermal organism will change as T_b_ changes, a relationship that has been described by a thermal performance curve (hereafter TPC) (Angilletta 2009). This curve is best captured by three parameters: a minimum critical temperature (CT_min_), which represents T_b_ below which performance is minimum, a maximum critical temperature (CT_max_), which represents T_b_ above which performance is also minimum, and an optimum temperature (T_opt_), which represents T_b_ at which performance is maximum. The curve rises gradually from CT_min_ to T_opt_ and then decreases gradually but rapidly to CT_max_. Recent analyses have shown that acclimation capacity of upper and lower thermal tolerances (i.e., CT_max_ and CT_min_, respectively) covaries positively with latitude (Stillman 2003; Somero 2010). This suggests that species at lower latitudes, which have evolved higher CT_max_, have achieved that at the expense of being less plastic (Stillman 2003). This in turn would suggest that lower latitude species are at a higher risk from climate change (Deutsch et al. 2008; Huey et al. 2009; Sinervo et al. 2010).

We evaluated this prediction in the northernmost population of *Pleurodema thaul* a small amphibian endemic to Chile and Argentina with a distributional range that spans more than 2500 km from the Atacama Desert (27°S) to Aysén (45°S) (Vidal et al. 2009) and from the Pacific coast up to 2700 m.a.s.l (Correa et al. 2007). As this population is located in a small oasis in the desert, clearly dispersal is not an option to warming temperatures. Therefore, compensation through behavior, plasticity, or evolutionary adaptation is a must in order to prevent a demographic collapse and extinction. In particular, we tested (i) whether there is physiological plasticity in the TPC of an ecological relevant trait to amphibians when acclimated to current and projected temperatures and (ii) whether there is an opportunity for behavioral thermoregulation in the landscape using high-resolution temperature data from biophysical models. In amphibians, most studies of plasticity under climate change have been focused on changes in breeding phenology (Table1 in Urban et al. 2014) as a consequence of past warming. Thus, this study is not only timely but also highly relevant as there is a need to understand how the physiological sensitivity of individuals might change under projected warming scenarios.

**Table 1:** Summary statistics for the thermal physiological traits (T_pref_ and resistance CT_min_ and CT_max_) and traits obtained from the TPC (T_opt_, V_max_, CT_max_)

	10°C	20°C	25°C
CRT_min_ (°C)	1.16 ± 0.90	−0.24 ± 1.15	0.00 ± 1.39
CTmin (°C)	−0.17 ± 0.11	−0.15 ± 0.10	−0.32 ± 0.31
T_pref_ (°C)	20.93 ± 4.62	21.17 ± 5.88	23.17 ± 6.03
T_opt_ (°C)	22.68 ± 2.67	25.98 ± 2.93	26.37 ± 3.70
CT_max_ (°C)	32.39 ± 1.60	34.46 ± 0.84	36.43 ± 1.92
CRT_max_ (°C)	36.73 ± 1.62	40.37 ± 2.83	41.14 ± 1.81
V_max_ (cm/sec)	8.46 ± 1.73	9.57 ± 2.58	11.41 ± 3.17

T_pref_, preferred temperature; CRT_min_, critical resistance minimum temperature; CRT_max_, critical resistance maximum temperature; T_opt_, optimal temperature; V_max_, maximum velocity at T_opt_; CT_max_, critical maximum temperature.

Critical resistance temperatures (CRT_min_ and CRT_max_) were determined as the environmental temperatures at which an individual lacked the ability to achieve an upright position within 1 min, while critical temperatures (CT_min_ and CT_max_) represent the point where the TPC intercepts the *x* -axis. See text for details on measurement and estimation methods. Data are presented as mean ± 1 SD.

## Materials and Methods

### Study organism and laboratory maintenance

Thirty-one individuals of *P*.  *thaul* were captured during April 2013 on two small ponds at Carrera Pinto (27°06′40.2″S, 69°53′44.3″W), an oasis in the Atacama Desert that is known to be the northernmost population of the species (Correa et al. 2007). All individuals were transported to the laboratory (Universidad Austral de Chile, Valdivia) within 2–3 days of capture.

Following capture, all animals were marked by toe clipping and maintained in the laboratory at a temperature of 20° ± 2°C and with a photoperiod 12D:12L. Animals were housed (*N*  = 5) in terrariums (length × width × height: 40 × 20 × 20 cm) provided with a cover of moss and vegetation and a small recipient filled with water. Individuals were fed once a week with mealworms (*Tenebrio molitor* larvae) and Mazuri® (St. Paul, Minnesota, USA) gel diets.

After 1 month at these conditions, individuals were acclimated for 2 weeks at 10°C, 20°C, and 25°C. We chose these acclimation temperatures because they are close to the mean annual temperatures during the breeding season (August – October, 10°C) and to the annual mean maximum temperatures (20°C) at Carrera Pinto. Finally, 25°C is close to the projected mean temperature under an A2 scenario at Carrera Pinto (IPCC 2007). For logistic reasons, animals were acclimated in series (i.e., first at 10°C then at 20°C and then at 25°). In order to remove any potential order effect from the signal (acclimation), we statistically incorporated the order of measuring as a random factor in all analyses. All physiological traits were measured after each acclimation with a 1-day rest between measurements. All individuals were in overall good health conditions during the whole experimental period as body mass did not show a negative (although also nor positive) trend with time (on log10 scale: *b*  = 0.00128; SE = 0.0009, CI 95: −0.0004–0.0030).

### Thermal performance curves

Performance was measured in a bioclimatic chamber as maximum jumping speed, a well-known trait of ecological relevance to amphibians (Navas et al. 2007). Individuals were cooled or heated to five or seven temperatures (acclimation at 10°C: 5, 10, 20, 26, and 29; acclimation at 20°C: 5, 10, 20, 26, 29, and 32; acclimation at 25°C: 5, 10, 20, 26, 29, 32, and 36°C) and were maintained for 1.30 h before each trial at the particular measurement temperature on individual hermetic cases with approximately 7 mm of water to standardize hydration levels.

**Figure 1:**
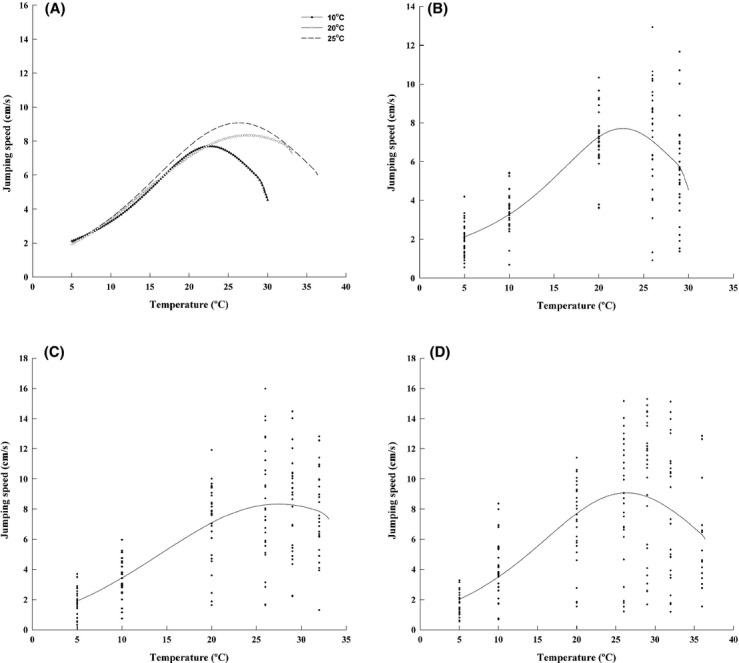
(A) Thermal performance curves under three different acclimation temperatures. See methods for details of estimation. Each point represents the mean value of all individuals at the measurement temperature. TPCs for acclimations at (B) 10°C, (C) 20°C and (D) 25°C.

Given that we had no previous knowledge of any performance curve for *P. thaul* particularly at high temperatures, we decided to follow a rather conservative approach in order to assure animals were in good conditions. Temperatures between 5°C and 29°C were applied in random order for each acclimation regime. After measuring performance at those five temperatures, we plot the data and evaluated whether the maximum performance was achieved or not. If not (i.e., acclimation at 20°C and 25°C), we run the trials again at 32°C. We repeated the procedure and run the trials again at 36°C for individuals acclimated at 25°C. It is clear in Fig.1 that individuals acclimated to 10°C have already achieved their maximum performance at 29°C and that individuals acclimated to 20° have achieved theirs at 32°C. In this sense, we are confident that estimated TPCs are not biased by the chosen T_b_s for each acclimation regime. Trials were run in a metallic lane of 75 cm (length) × 12 cm (width) × 20 cm (height) within the bioclimatic chamber. We confirmed that each individual reached the target body temperature (T_b_) registering dorsal T_b_ using a UEi INF155 Scout1 infrared thermometer (see below MODEL CALIBRATION). The infrared thermometer was gently pressed on the frog to obtain dorsal T_b_. Each individual was motivated to jump-run by gentle touching it on the dorsal–caudal body region until it reached the other end of the lane and was allowed to explore the line for a couple of minutes before registering velocity. Performance was measured as the time needed for an individual to reach the end of the lane and was measured twice per individual at each temperature, with measurements 1 h apart between them. The individual performance at that temperature was the average of the two. Measurements at different temperatures were taken every 48 h. Body size was obtained before and after each trial using a Shimadzu TX323L (Shimadzu Corp. Kyoto, Japan) electronic balance. Body length was obtained using a digital caliper as all velocities were corrected by each individual's length.

### Thermal physiology

Upper (CRT_max_) and lower (CRT_min_) critical resistance temperatures were determined as the environmental temperatures at which we observed a loss of righting response within 1 min (e.g., Bacigalupe et al. 2007). Each individual was placed in a small chamber with several respiration holes in a metal box which was inside a thermoregulated bath (WRC-P8, Daihan, Korea) at 30°C (CRT_max_) and 5°C (CRT_min_) for 15 min, after which we increased (or decreased) the temperatures at a rate of 0.8°C per minute (Rezende et al. 2011). A similar small chamber with a HOBO (Onset Computer Corporation, Bourne, Massachusetts, USA) data logger (Onset, Model U23-003) was used to evaluate Ta inside the chamber. Every minute or at every change in 1°C, we turned upside down the chamber and observed whether the animal was able to return to the upright position. When an animal was unable to achieve an upright position within 1 min, we let it recover at ambient temperature (CRT_min_) or for 30 min in a box with ice packs (CRT_max_). Body size was obtained before each trial using a Shimadzu TX323L electronic balance.

Preferred temperature (T_pref_) was determined individually in five open-top terraria (length × width × height: 85 × 12 × 30 cm) each with gardening organic soil and a thermal gradient produced by an infrared lamp overhead (250°W) on one end and ice packs on the other. The lamp's height was adjusted to provide a temperature of approximately 30°C at the soil level. The temperature gradient was between 10°C and 30°C. The soil was moistened at the beginning of each trial to prevent the desiccation of the frogs. Five individuals were placed at the center of each one of the terraria, and 45 min later, we registered T_pref_ as the dorsal T_b_ using a UEi INF155 Scout1 infrared thermometer. Body size was obtained before each trial using a Shimadzu TX323L electronic balance.

For all acclimation temperatures, the different traits were measured in the following order: CRT_min_, T_pref_, TPC, and CRT_max_.

### Operative temperatures in the field

Operative temperature (T_e_) models were made of agar (agar E406) to ensure they have the same size, and the same thermal and evaporative properties of live frogs (Navas and Araujo 2000). Eight frog models were deployed during the current breeding season (October 2013) at the two ponds in Carrera Pinto (four per pond) each in a specific combination of sun or shade and wet (model placed on wet soil) or dry (model placed on dry soil) conditions. Each model had incorporated a HOBO data logger (Onset, Model U23-003), and temperature was registered every 5 min for 24 h. Each model was used for up to approximately 4–5 h during the day and 10–12 h during the night. Each model was weighted with a DigiWeigh DW-100AS balance before and after being used in order to have an estimate of the rate of water loss.

### Model calibration

To calibrate dorsal T_b_ against cloacal T_b_, 25 individuals were measured at 0, 5, 10, 15, 20, 25, and 30°C using a thermoregulated bath (WRC-P8, Daihan, Korea). Each individual was placed within a small chamber during 60 min before determining T_b_ with the infrared thermometer and a dual-channel thermocouple thermometer (Cole-Parmer (Vernon Hills, Illinois, USA) EW-91210-01). The number of individuals in each temperature ranged from 5 to 11, and some individuals were used in more than one temperature. Data for each temperature were averaged for analyses. Cloacal and dorsal T_b_ closely followed environmental temperature (T_a_ − cloacal T_b_: *r*_*P*_ = 0.98, *t * = * * 10.26_[5]_, *P*  < 0.001, T_a_ − dorsal T_b_: *r*_*P*_ = 0.98, *t * = * * 12.17_[5]_, *P*  < 0.001). Furthermore, dorsal T_b_ was closely associated to cloacal T_b_ (*r*_*P*_ = 0.99, *t * = * * 20.79_[5]_, *P*  < 0.001).

In order to determine whether models T_e_ represent T_b_ of live animals accurately, we measured T_b_ of individuals at different times and in the four different combinations of sun, shade, wet, and dry over the course of 1 day. The agar models T_e_ (mean: 18.56 ± 2.07 SE, *N*  = 12) accurately reflected frog T_b_ (mean: 18.48 ± 1.64 SE, *N*  = 12) and both were statistically indistinguishable (*F*_1,22_ = 0.001, *P * = * * 0.976).

### Statistical analyses

Thermal performance curves were fitted through several functions (e.g., Gaussian, Lorentzian, Weibull), and the best fit was obtained using the Akaike's information criterion (Anderson 2008). TPCs for each individual were described in terms of the optimal temperature (T_opt_), the maximal performance (V_max_), and the lower and upper critical limits of temperature at which the performance was zero (i.e., the point where the curve intercepts the *x* -axis, CT_min_ and CT_max_) (Angilletta 2009). We used the Table Curve2D curve-fitting software (version 5.01; Systat Software (San Jose, California, USA), Inc.) for model fitting. Individual TPC parameters (V_max_, T_opt_, CT_min_, and CT_max_) were extracted from the best models.

Thermal physiological traits (T_pref_ and resistance CRT_min_ and CRT_max_) and traits obtained from the TPC (CT_min_, T_opt_, V_max_, and CT_max_) were analyzed using a mixed modeling approach, as we have three repeated measures on the same individual. The effect of acclimation temperature (fixed effect) was evaluated through confidence intervals computed from the likelihood profile (Bates et al. 2013). The order of measuring was included in all analyses as a random factor. Traits were log10-transformed to meet normality assumptions. Therefore, results are presented as a CI 95 for mean differences based on log10-transformed data. Log10-transformed body mass was used as a covariate for maximal performance and CRT_min_.

For each frog model at each pond and at each combination of dry–wet and sun–shade, we averaged the T_e_ between 6:00 and 20:00. We carried out a two-way ANOVA to evaluate the joint effects of both factors on T_e_, T_e-max_ (maximum value of T_e_ in that particular combination of factors) and water loss. Analyses were carried out using R 2.15.0 (R Core Team 2013).

## Results

### Thermal performance curves

Summary statistics for the thermal physiological traits (T_pref_, CRT_min,_ and CRT_max_) and traits obtained from the TPC (CT_min_, T_opt_, V_max_, and CT_max_) are presented in Table1.

The best-fit models describing the thermal performance curves for each acclimation temperature (Table2) showed the typical left-skewed shape (Fig.1). T_opt_ increased from acclimation at 10°C to acclimation at 20°C (CI 95 for mean differences: 0.029–0.084) but not from acclimation at 20°C to acclimation at 25°C (CI 95 for mean differences: −0.033–0.022) (Fig.1). As T_opt_ shifted to the right, the upper temperature limits were also shifted (Fig.1): CT_max_ increased from acclimation at 10°C to acclimation at 20°C (CI 95 for mean differences: 0.017–0.034) and also from acclimation at 20°C to acclimation at 25° (CI 95 for mean differences: 0.015–0.032). The critical minimum temperature did not change between acclimation at 10°C and acclimation at 20°C (CI 95 for mean differences: −0.076–0.124), but it decreased from acclimation at 20°C to acclimation at 25°C (CI 95 for mean differences: −0.271 to −0.068). Finally, maximal performance was not different between acclimation at 10°C to 20°C (CI 95 for mean differences: −0.007–0.103) nor it was different from acclimation at 20°C to acclimation at 25°C (CI 95 for mean differences: −0.110–0.024) (Fig.1 and Table2). In overall, TPCs increased their breath under warmer acclimations by shifting their upper limits.

**Table 2:** Comparison of functions used to describe the thermal performance curves of *Pleurodema thaul* under different acclimation temperatures using Akaike's information criterion (AIC). The function with the lowest AIC was the one chosen as the best

Acclimation	Function	K	AIC	*λ*_i_	*w*_i_	*r*^2^
10°C	**Lorentzian**	3	−57.22	0	0.75	0.992
	Logistic	3	−54.80	2.41	0.23	0.990
	Gaussian	3	−50.07	7.14	0.02	0.985
10°C	**Gaussian**	3	−61.37	0	0.99	0.998
	Logistic	3	−51.88	9.49	0.01	0.995
	Lorentzian	3	−35.22	26.15	0	0.981
10°C	**Gaussian**	3	38.47	0	0.48	0.967
	Logistic	3	38.52	0.04	0.47	0.969
	Lorentzian	3	43.20	4.73	0.05	0.966

K, number of parameters in the function; *λ*_i_, difference between a given model's AIC and the lowest AIC; *w*_i_, Akaike's weight.

Models in boldface were selected for obtaining the individuals parameters.

### Thermal physiology

T_pref_ was not different between acclimation at 10°C and acclimation at 20°C (CI 95 for mean differences: −0.059, 0.053) or between acclimation at 20°C and acclimation at 25°C (CI 95 for mean differences: −0.098, 0.016) (Table1). On the other hand, resistance thermal maximum (CRT_max_) increased between acclimation at 10°C and acclimation at 20°C (CI 95 for mean differences: 0.028–0.053) but not from acclimation at 20°C to acclimation at 25°C (CI 95 for mean differences: −0.021–0.004). Similarly, the critical resistance thermal minimum (CRT_min_) decreased from acclimation at 10°C to acclimation at 20°C (CI 95 for mean differences: −0.291 to −0.124) but not from acclimation at 20°C to acclimation at 25°C (CI 95 for mean differences: −0.117–0.053).

### Operative temperatures in the field

**Figure 2:**
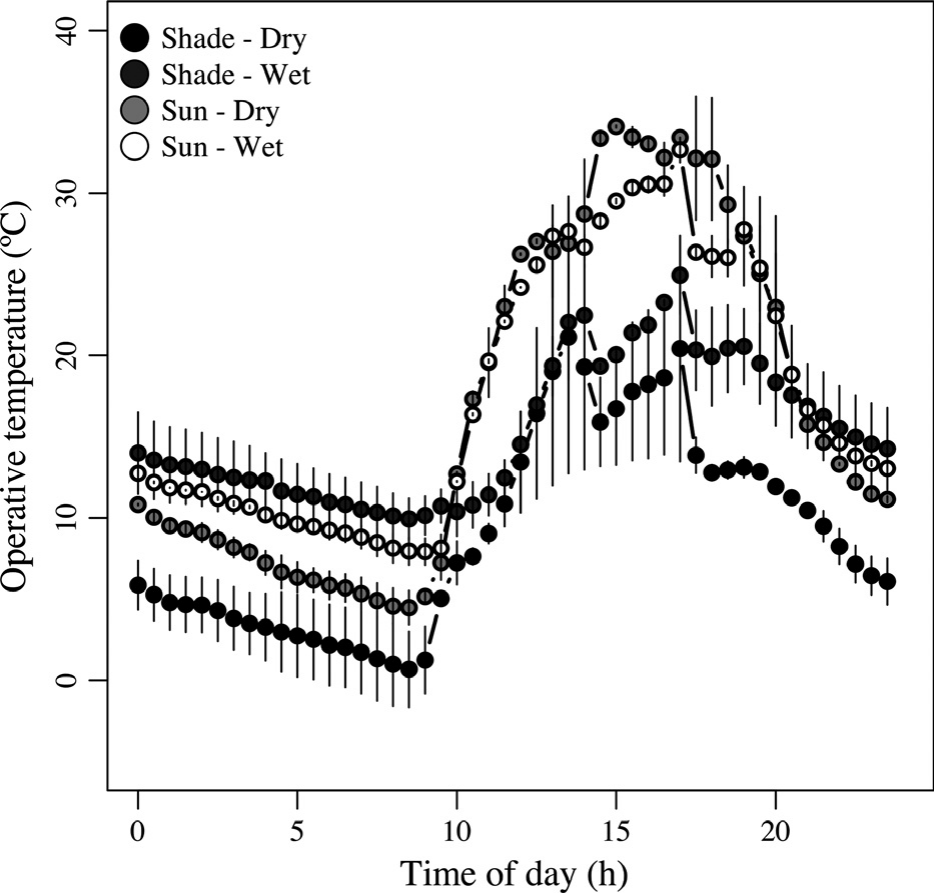
Temporal distribution of T_e_s among habitat types (shade–sun) and conditions (dry–wet). Each point is the average (±1 SD) between two frog models for a 30-min interval.

The temporal distribution of T_e_ was different between sun–shade and dry–wet conditions (Fig.2). Daytime (07:00–20:00) mean T_e_ was only affected by sun exposure (*F*_1,5_ = 23.49, *P * = * * 0.005), but not by dry–wet conditions (*F*_1,5_ = 1.63, *P * = * * 0.258) or their interaction (*F*_1,4_ = 6.37, *P * = * * 0.065). Mean T_e_ during daytime was 7.83°C higher in the sunshine than in the shade. A similar pattern was observed for T_e-max_. Daytime T_e-max_ was 11.13°C higher in the sunshine than in the shade (*F*_1,5_ = 10.46, *P * = * * 0.023) and was not affected by dry–wet conditions (*F*_1,5_ = 0.07, *P * = * * 0.806) or their interaction (*F*_1,4_ = 0.866, *P * = * * 0.405). Finally, daytime rate of water loss was 0.686 grams/h smaller under wet than under dry conditions (*F*_1,5_ = 17.75, *P * = * * 0.008) and 0.403 grams/h higher in the sunshine (*F*_1,5_ = 6.11, *P * = * * 0.056). Daytime water loss was not affected by the interaction of both factors (*F*_1,4_ = 0.847, *P * = * * 0.410).

**Figure 3:**
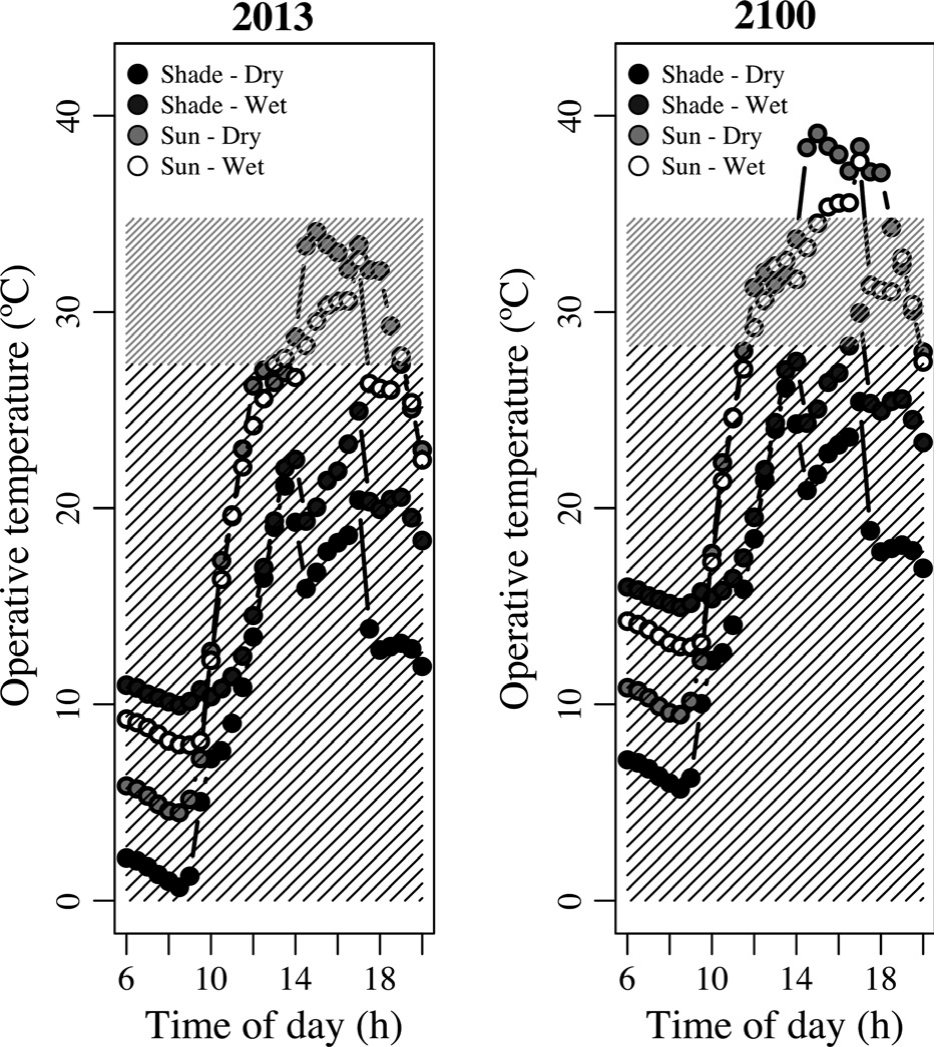
Temporal distribution of daytime (06:00–20:00) T_e_s for current (2013) and projected conditions (2100). Each point is the average between two frog models for a 30-min interval. Temperature projections of 5°C are based on IPCC estimates under an A2 scenario. Shaded regions represent thermal limits between CT_min_ and T_opt_ (black lines) and between T_opt_ and CT_max_ (gray lines) were obtained from the TPC of individuals acclimated to 20°C (2013) and 25°C (2100) (see Table1). Those limits represent the point where the TPC intercepted the *x* -axis. See text for details on estimation.

We also evaluated whether climate warming would reduce the temporal availability of T_e_s within the limits of the thermal performance curves, assuming that T_e_ scales linearly with T_a_ (Bakken 1992; equation 1). Under current climatic conditions, frogs would not be exposed to T_e_s outside its tolerance limits (Fig.3). On the other hand, under an extreme warming of 5°C, behavioral thermoregulation is a must: 25% of the time T_e_ exceeds CT_max_ in the sun–dry and almost 15% of the time in the sun–wet patches.

## Discussion

When dispersal is not an option to evade warming temperatures, compensation through behavior, plasticity, or evolutionary adaptation is essential to prevent extinction. In this work, we evaluated whether there is physiological plasticity in the TPC of maximum jumping speed in individuals acclimated to current and projected temperatures and whether there is an opportunity for behavioral thermoregulation in the desert landscape where inhabits the northernmost population of the frog *P. thaul*. Our results indicate that individuals acclimated to 20°C and 25°C increased the breath of their TPCs by shifting their upper limits. In addition, even when dispersal is not possible for this population, the landscape is heterogeneous enough to offer opportunities for behavioral thermoregulation. In particular, under current climatic conditions, behavioral thermoregulation is not compulsory as available T_es_ are encompassed within the population TPC limits. However, for severe projected temperatures under climate change, behavioral thermoregulation will be required in the sunny patches during some hours of the day.

The physiological impact of climate warming depends mostly on an organism's T_b_ at the onset of warming relative to T_opt_ (Huey et al. 2012). As field T_b_ was accurately reflected by the biophysical model's T_e_ (see Results), we assume that T_b_ throughout the day is a close approximation to registered T_e_s. Thus, under current conditions, the average daytime T_e_ is lower than T_opt_ in all combinations of sun, shade, wet, or dry patches (mean: shade–wet = 16.4°C; shade–sun = 11.2°C; sun–wet = 20.9°C; sun–dry = 21.4°C).

Nevertheless, a close inspection at Fig.2 shows that mean T_e_ does not really reflect the temperature being experienced at all times by the frogs as this depends on the particular patch being observed (Kearney et al. 2012; Scheffers et al. 2014). While shade conditions have T_e_s below T_opt_ during all day, sun patches are already at or beyond T_opt_ for much of the daytime. In this context, things get harsher under a projected warming of 5°C. Assuming that T_e_ scales linearly with environmental temperature (Bakken 1992), by 2100, behavioral thermoregulation will have to be compulsory to buffer T_b_ at least 25% of the time in the sun–dry and almost 15% of the time in the sun–wet patches (Kearney et al. 2009; Logan et al. 2013). Therefore, during those times that T_e_ surpassed the critical thermal limits, frogs have to rely on shaded patches to avoid overheating or be more frequently in the water or move in and out of shade or water to stay cool. Although there might be some limits on amphibian behavioral thermoregulation (Tracy 1976), we have some preliminary observations for this locality that suggest that frogs are already using behavior to thermoregulate (i.e., diving into the ponds during the hottest hours). Furthermore, there is an urgent need to understand the dynamics of T_b_ under conditions above CT_max_ as survival is not only determined by the intensity of the thermal stress but also determined by its duration (Rezende et al. 2014).

*Pleurodema thaul* has a wide distributional range in latitude that covers an extensive number of biomes, from the Atacama Desert to the Chilean temperate rainforest (Vidal et al. 2009; Correa et al. 2007). This also means that patterns of geographic variation are highly likely to occur, as has been found for reproductive and life-history traits (Iturra-Cid et al. 2010). Interestingly, the thermal physiology of the species is barely known and just a single study has evaluated the effect of latitude on physiological traits (*Myriam Iturra-Cid, Marcela Vidal, Leonardo D. Bacigalupe and Juan C. Ortiz, unpublished results*). In particular, this study found a strong latitudinal pattern in CRT_max_ and in its acclimatory capacity (10°C–20°C), suggesting that populations from lower latitudes are already living closer to their thermal limits. As the population studied here (i.e., Carrera Pinto) is 330 km further north than the northernmost one in the mentioned study, we expected the pattern to be confirmed. Although our results agree with this, there were also some differences. CRT_max_ was even higher in animals acclimated to 20°C (mean ± SD: Carrera Pinto = 40.4°C ± 2.8°C; La Serena = 38.5°C ± 0.8°C; t_[35]_ = 3.37, *P * < * * 0.05). However, acclimatory capacity of CRT_max_ was not reduced (ΔCRT_max_ [20°C–10°C]: Carrera Pinto = 3.6°C; La Serena = 1°C). Two reasons may account for this. First, Carrera Pinto is almost at 1800 m.a.s.l., and thus, the lower CTR_max_ in individuals acclimated at 10°C may reflect the colder temperatures at which they are exposed in their environment. Second, although both studies used the same ramping protocol to estimate thermal limits, in the previous study, CRT were measured from the acclimation temperatures (10°C and 20°C), while here (based on information provided by that study), we started at 30°C (CRT_max_) and 5°C (CRT_min_). Therefore, in the first study, animals were longer under stress and thus, limits might have been underestimated (Rezende et al. 2014). Nevertheless, we consider that measured CTR_max_ in individuals acclimated to 20°C in Carrera Pinto is not an artifact as animals are exposed to high temperatures all the year, and it is known that thermal limits are more responsive to thermal extremes than mean temperatures (Huey and Kingsolver 1993).

In overall, our results suggest that this population of *Pleurodema thaul* will be able to endure the worst projected scenario of climate warming as it has not only the physiological capacities but also the environmental opportunities to regulate its body temperature behaviorally. Nevertheless, it should be noted that we have measured the plasticity of only one trait and in just one life stage (Kingsolver et al. 2011). Although *P. thaul* ’ larvae strictly inhabit water bodies, during the non-reproductive period, adults can move around and are usually found under rocks or logs. However, at Carrera Pinto (the oasis from where the population for this study came from), adults are during all year very near to the water bodies. In any case, although other ecological and physiological traits might also be plastic, their thermal sensitivities might be different (Angilletta 2009) and they might be also different between different life stages and thus, only further work in other traits and stages might disentangle this. Our study also highlights the importance of considering microhabitats when evaluating the real impact warming will have on a population and thus its vulnerability (Kearney et al. 2009; Scheffers et al. 2014; Logan et al. 2013; Kearney 2013). This might seem a daunting task at first, but surely the rewards in terms of better predictions and management for conservation purposes will compensate the effort invested. It is also important to note that the thermal environment of frogs might be more complex than just T_a_ (Tracy 1976), and thus, our results should be interpreted with caution in that sense. With that caveats in mind, we still consider our results show a strong signal on the importance of incorporating performance physiology data with relevant organismal processes (e.g., phenotypic plasticity) to evaluate the actual risk of extinction of a population (Gerick et al. 2014).
